# Increased apoptosis in late-developing *in vitro* fertilized bovine blastocysts decreases successful pregnancy

**DOI:** 10.5713/ab.24.0454

**Published:** 2025-01-15

**Authors:** Tae-Gyun Kim, Yong-Ho Choe, Sung-Ho Kim, Sang-Yup Lee, Min Jang, Sung-Ho Yun, Seung-Joon Kim, Sung-Lim Lee, Won-Jae Lee

**Affiliations:** 1 College of Veterinary Medicine, Kyungpook National University, Daegu 41566, Korea; 2 College of Veterinary Medicine, Gyeongsang National University, Jinju 52828, Korea; 3 Bovivet, Gumi 39133, Korea

**Keywords:** Apoptosis, Bovine Blastocyst, Embryo Developmental Speed, Embryo Transfer, Hanwoo, Pregnancy Rate

## Abstract

**Objective:**

Pregnancy in cattle after embryo transfer (ET) is influenced by several factors, including embryo quality. Therefore, preparing high-quality embryos with the greatest developmental potential is essential for achieving a successful pregnancy after ET. Meanwhile, blastocysts produced by *in vitro* fertilization (IVF) procedure have different developmental speed during *in vitro* culture (IVC) and they exhibited different competence in the establishment of pregnancy.

**Methods:**

This study aimed to identify the comparative features of early-, mid-, and late-developing bovine IVF blastocysts, when they first appeared at Day 7, 8, and 9 during IVC, respectively. In addition, the correlations between their molecular features and pregnancy ability were analyzed.

**Results:**

The results showed no difference in the morphological characteristics, including total cell count and diameter, between the Day 7, 8, and 9 blastocysts. However, the pregnancy rate post-ET was significantly different between the groups at 51.7%, 36.7%, and 17.8% for Day 7, 8, and 9 blastocysts, respectively. During early embryo development, late-developing blastocysts demonstrated a reduced cell count in the inner cell mass and decreased expression of the early embryo developmental genes (*Oct4* and *Sox2*) compared with the early- and mid-developing blastocysts. In addition, the number of apoptotic cells and apoptosis-related gene expression (increased *Bax* and decreased *Bcl2*) gradually elevated from the Day 7 to Day 9 blastocysts. However, there was no difference in mitochondrial activity and mitochondria-relevant gene expression (*Tfam* and *Cox1*) between the groups. Correlation analysis identified a significantly negative correlation between the pregnancy rate and the blastocysts’ degree of apoptosis, indicating that the low pregnancy ability of late-developing blastocysts was mainly caused by increased apoptosis.

**Conclusion:**

This study’s results may contribute to the field of animal biotechnology by assisting in establishing an improved strategy for bovine ET with IVF embryos.

## INTRODUCTION

Embryo transfer (ET) has developed considerably in the animal biosciences since the first successful case was reported in 1891 using rabbits [1]. This is especially true for livestock reproduction, including cattle, in which ET is technically used as a tool for the recovery of infertile female animals (repeat-breeders), reproductive disease control, shortened delivery intervals, and convenience during the import and export of genetic resources [1,2]. The breeding strategy with artificial insemination delivers paternal genetic information only; in contrast, ET is capable of accelerating to produce genetically superior offspring with genetic information from both bull and dam. In addition, several articles have reported higher pregnancy rates in bovine ET recipients compared with artificially inseminated females, especially under heat-stress conditions during summer or in repeat breeders [1,3]. Furthermore, cattle pregnancy rates post a single artificial insemination treatment have reportedly declined globally by 0.45% annually and ET has been suggested as an alternative to overcome this decrease [4]. Therefore, the advantages of using ET in cattle breeding have increased its popularity, with a reported 1,000,000 procedures performed during 2021 using *in vivo*-produced and *in vitro*-fertilized embryos [5].

Success rates in the production yield of bovine blastocysts using *in vitro* fertilization (IVF) are reportedly around 30% and pregnancy rates post-ET vary depending on the experimental conditions but are generally between 30% and 65% [3,6,7]. Many factors influence successful pregnancy rates after ET, including the embryo’s quality, the ET technician’s skill, the use of either a frozen or fresh embryo, the quality of the corpus luteum (CL), management of the recipient, the season of conducting the ET, and the type of recipient, for example, beef vs dairy cattle or heifer vs cows [8,9]. In case of IVF embryo production, the formation of blastocysts post-IVF and during *in vitro* culture (IVC) is not simultaneous but varies between 6.5 and 10 days, which can influence the embryo’s quality. Of note, embryos may exhibit different competence in the establishment of pregnancy depending on their developmental speed. For instance, early-developing IVF blastocysts (first formed at Day 6 to 7 of the IVC) have a higher pregnancy rate in Holstein cows compared with late-developing blastocysts (first formed at Day 7 to 8 of the IVC) [8,10]. Similarly, in Hanwoo (*Bos taurus coreanae*) IVF embryos, the blastocysts that first appeared on Day 7 of the IVC exhibited a higher pregnancy rate compared with blastocysts presented between Day 8 and 9 of the IVC [6].

Therefore, extensive studies using several analytical methods have evaluated the quality of embryos to improve pregnancy rates [4,9]. Mortality or developmental competence of embryos is coupled with altered gene expression during the early developmental period [11]. To assess embryo quality, early embryo developmental gene (*Oct4*, *Sox2*, and *Cdx2*) expression studies have been widely conducted. The *Oct4* gene is crucial in pluripotency regulatory networks in several species; it supports indefinite self-renewal and the capacity to differentiate toward several lineages in bovine blastocysts and is expressed in their inner cell mass (ICM) and trophectoderm (TE) cells [12]. In addition, the *Sox2* is a key factor for early embryo development and maintaining pluripotency in bovine blastocysts and is expressed from the morula stage only and is restricted to the ICM area of the blastocyst [12]. Furthermore, the *Cdx2* plays a pivotal role in the segregation of the ICM and TE lineages at the blastocyst stage by repressing *Oct4* expression in the TE [13].

Several authors have reported that programmed cell death, called apoptosis, is unavoidable during an embryo’s early development to counterbalance its cell survival and proliferation [14,15]. The morphology of cells under apoptosis presents a condensed cytoplasm, swollen endoplasmic reticulum, and cytoplasmic blebbing. In addition, apoptotic cell death is involved with the up-regulation of pro-apoptotic genes (e.g., *Bax* and *Bak*) and the down-regulation of anti-apoptotic genes (e.g., *Bcl2*) [15].

Mitochondria also play a vital role in early embryo development by producing adenosine triphosphate (ATP) in the oocytes, which supplies energy for fertilization and pre-implantation development. Notably, the homogenous distribution of mitochondria in the immature oocyte stage changes to a heterogeneous pattern aggregated around the nucleus during oocyte maturation (meiosis); failure of this redistribution induces oocyte arrest, embryo developmental block, and lowering the quality of the embryo caused by an improper energy supply [4,16]. Furthermore, a mitochondrial activity gene expression study that investigated transcriptional activation in developing bovine embryos until the morula stage found that *Cox1* and *Tfam* were *de novo* synthesized, depending on the developmental stages [17].

As previously mentioned, various factors may influence the success of ET until full-term delivery in cattle; therefore, every step from the production of the IVF embryos to the ET is crucial to the success of offspring production. Among them, selecting a high-quality embryo with the best developmental potential is a key factor in overcoming several of ET’s obstacles [5,8,9]. Currently, blastocysts’ morphological assessment is regarded as the ‘gold standard’ for selecting promising embryos for pregnancy. However, even if the embryos are of an excellent grade based on the morphological criteria, this does not necessarily reflect its intrinsic embryonic competence. In addition, although it has been reported that late-developing embryos have an inferior pregnancy ability, a comparative study of their molecular characteristics has yet to be conducted. Therefore, this study aimed to compare the molecular characteristics, including early embryo developmental ability, apoptosis rate, mitochondrial membrane potential, and gene expression, with the pregnancy rate between bovine IVF blastocysts with different developmental speed, that is, early-, mid-, and late-developing blastocysts first appeared at Day 7, 8, and 9 during IVC.

## MATERIAL AND METHODS

### Ethical statements

All experimental procedures in animal were approved by the Institutional Animal Care and Use Committee at Kyungpook National University (approval number: 2023-0372).

### Chemicals and media

All chemicals and reagents were obtained from Thermo Fisher Scientific (Waltham, MA, USA) unless otherwise specified.

### *In vitro* production of bovine embryos

Bovine ovaries were obtained from a local slaughterhouse about an hour away from the laboratory, and transported to the laboratory at 37°C in a portable constant-temperature water bath. The ovaries were washed three times with phosphate-buffered saline (PBS) on arrival at the laboratory. Subsequently, cumulus–oocyte complexes (COCs) were collected from the ovarian follicles (3 to 8 mm in diameter) into glass tubes using a 19 gauge needle; only COCs that had compact layers of cumulus cells and contained homogeneous cytoplasm were selected. The COCs were *in vitro* matured using Medium 199 supplemented with 5 μg/mL follicle-stimulating hormone, 10 IU/mL luteinizing hormone, 1 μg/mL 17β-estradiol, 10% fetal bovine serum, 0.2 mM sodium pyruvate, and 0.05 mg/mL gentamycin in an atmosphere of 5% CO_2_ in humidified air at 38.5°C for 22 to 24 hours. The mature COCs were prepared in a drop culture system (10 COCs per drop) with Tyrode albumin lactate pyruvate media supplemented with 20 μg/mL heparin. The frozen semen was thawed in a water bath at 35°C, washed twice with Tyrode albumin lactate pyruvate media, and centrifugated at 1,000×g for 10 minutes. Then, motile sperms were collected by the swim-up method for an hour. The prepared COCs in drops were fertilized with a final sperm concentration of 2×10^6^ sperm/mL in an atmosphere of 5% CO_2_ in humidified air at 38.5°C for 18 hours. Day 0 in the embryo’s development corresponded to the time of sperm insemination. The cumulus cells around presumptive zygotes were denuded by EZ-Grip with EZ-Tip (CooperSurgical Fertility Solutions, Ballerup, Denmark) and cultured with synthetic oviductal fluid media supplemented with 5 mg/mL bovine serum albumin and 0.05 mg/mL gentamycin (IVC media) in an atmosphere of 5% each of O_2_ and CO_2_ in humidified air at 38.5°C until the appearance of blastocysts between days 7 and 9 post IVF; during the IVC, cleaved zygotes were selected at Day 3 and fresh media was introduced at Day 3 and 5. On the first day that stage 6 and 7 blastocysts, according to the International Embryo Technology Society’s criteria, appeared during the IVC at days 7, 8, or 9 post-IVF, they were collected for Day 7, 8, and 9 blastocysts as early-, mid-, and late-developing blastocysts, respectively [9]. The IVF blastocysts with different developmental speed were obtained via 14 replicates using the same method. This study strived for objective results by randomly allocating the blastocysts derived from a batch of each IVF procedure for ET and molecular assays concurrently; once the IVF blastocysts of each group per batch were allocated to ET, those remaining were sampled by snap-freezing with liquid nitrogen for gene expression studies or fixation with 4% paraformaldehyde for morphological observation, differential staining for early embryo development examination, apoptosis-related assays, and checking the mitochondrial membrane potential.

### Embryo transfer and pregnancy diagnosis

ET were conducted from early to late summer; the maximal atmospheric temperature of the days ranged between 27.4°C and 35.2°C. The IVF blastocysts were transferred into recipients of another breed (Holstein heifer, N = 198, average age approximately 1.5 years, and a body condition score ranging between 2.5 and 3.0). The estrus cycles of the recipients were synchronized with an IVF blastocyst production schedule employing the Ovsynch protocol (a gonadotropin-releasing hormone analog injection [Buserelin, buserelin acetate; Unibiotech Co., Ltd., Anyang, Korea], followed by a prostaglandin F2α analog injection [Repromate, cloprostenol sodium; Unibiotech Co., Ltd] after 7 days, and then a second treatment with gonadotropin-releasing hormone analog 48 hours later); the onset of estrus in the recipients was considered as day 0. A day before the ET, the CLs of the recipients were evaluated by transrectal ultrasonography to select those with a healthy and mature CL (≥15 mm). Finally, ET was conducted using 166 recipients on days 7 post the Ovsynch protocol by a skilled veterinarian (Day 7 blastocysts: total N = 90 for 14 ET trials, Day 8 blastocysts: total N = 40 for 12 ET trials, Day 9 blastocysts: total N = 36 for 8 ET trials). The Day 7, 8, and 9 blastocysts were gently packed separately in 0.25 mL mini straws (IMV Technologies, L’Aigle, France) with IVC media. The straws were heat-sealed, horizontally laid in a thermos set at 38°C, and carefully transported to the recipients’ farm. The blastocysts were non-surgically transferred to the recipient’s most distal point of the uterine horn ipsilateral to the CL using an ET gun. The early pregnant diagnosis was first conducted 30 days post-ET, using an iScan with a 7.5 MHz linear transducer (DRAMIŃSKI, Sząbruk, Poland) to check for the presence of a gestational vesicle; this is a hypo-echogenic embryo inside a non-echogenic area of the uterus, which indicates pregnancy. A second pregnancy diagnosis was conducted at 60 days post-ET; the disappearance of a fetus or fetal heartbeat, and/or the absence of fetal fluids was diagnosed as a pregnancy loss [9].

### Morphological observation of the bovine blastocysts

To evaluate the diameter and total cell number of each blastocyst, the fixed blastocysts were washed twice with PBS containing 0.1% polyvinyl alcohol (PBS-PVA) and then stained with 4′,6-diamidino-2-phenylindole (DAPI) for 5 minutes at room temperature (RT). Then the stained blastocysts were mounted on slides with Vectashield mounting medium (Vector Laboratories Inc., Newark, CA, USA) and a coverslip, and the blue fluorescence signal was observed at a 461 nm wavelength (Day 7 blastocysts: total N = 14, Day 8 blastocysts: total N = 14, Day 9 blastocysts: total N = 10).

### Immunofluorescence for differential staining of the bovine blastocysts’ inner cell mass

The transcription factor *Sox2* is specifically expressed in bovine blastocysts’ ICM; *Sox2* gives rise to the fetus’ definitive structures and is regarded as a specific marker for counting the number of cells in the ICM by differential staining of the blastocysts [12]. First, the fixed blastocysts were washed twice with PBS-PVA, and permeabilized with PBS supplemented with 10% fetal bovine serum and 0.1% Triton X-100 for 1 hour at RT. Then, the fixed blastocysts were incubated with an anti-*Sox2* monoclonal antibody (dilution factor: 1:500 in PBS-PVA; Cell Signaling Technology Inc., Beverly, MA, USA) for 4 hours at RT on a rocking shaker. After washing the fixed blastocysts with PBS-PVA twice, they were treated with PBS-PVA containing the secondary antibody, Alexa Fluor 555 donkey anti-goat IgG antibody (dilution factor: 1:3,000 in PBS) for an hour at RT on a rocking shaker. Finally, counterstaining with DAPI was conducted for 5 minutes at RT, which was followed by mounting the samples on slides with Vectashield mounting medium and a coverslip (Day 7 blastocysts: total N = 14, Day 8 blastocysts: total N = 14, Day 9 blastocysts: total N = 10). Using a fluorescence microscope, images for the blue (DAPI-positive cells, nucleus) or red (*Sox2*-positive cells, ICM) fluorescence signals at a 461 or 590 nm wavelength, respectively, were obtained. The DAPI-positive cells were regarded as a blastocyst’s total cell number and the *Sox2*-coexpressing DAPI-positive cells were deemed as their ICM. The cell number of the TE was calculated by subtracting the ICM number from the total cell number.

### Measurement of the bovine blastocysts’ apoptosis

To assess the cellular apoptosis level of Day 7, 8, and 9 blastocysts, terminal deoxynucleotidyl transferase dUTP nick end labeling (TUNEL) staining was used to determine the deoxyribonucleic acid (DNA) fragmentation level (cellular apoptosis) in the bovine blastocysts using an *in situ* cell death detection kit (Roche Diagnostics International Ltd., Rotkreuz, Switzerland) following the manufacturer’s instruction. The fixed blastocysts were washed with PBS-PVA, permeabilized with 0.1% sodium citrate containing 0.5% Triton X-100 for an hour at RT, and then incubated in the dark with TUNEL solution for an hour at 38°C. After washing twice with PBS-PVA, the blastocysts were counterstained with DAPI for 5 minutes. After mounting the samples on a slide with Vectashield mounting medium and a coverslip, fluorescent images were obtained using a fluorescence microscope under blue (DAPI, nucleus) or green (TUNEL, apoptotic bodies) fluorescence signals at 461 nm or 520 to 527 nm wavelengths, respectively (Day 7 blastocysts: total N = 14, Day 8 blastocysts: total N = 14, Day 9 blastocysts: total N = 10). The number of apoptotic cells was calculated as a percent (%) of the total cell number.

### Mitochondrial membrane potential assessment of the bovine blastocysts

To analyze mitochondrial membrane potential, which changes depending on the embryo stages [18], the fixed blastocysts were washed twice with PBS-PVA and incubated with 5 μg/mL 5,5′,6,6′-tetrachloro-1,1′,3,3′-tetraethylbenzimidazolyl-carbocyanine iodide (JC-1 dye) for 30 minutes at 38°C. This fluorochrome dye reacts with monomers in low membrane potential mitochondria or aggregates to produce high membrane potential mitochondria, which are observed using green or red fluorescence signals, respectively. The incubated blastocysts were mounted on slides with Vectashield mounting medium and a coverslip, and observed for the green (monomers) or red (aggregates) fluorescence signals at a 520–527 or 590 nm wavelength, using a fluorescence microscope, respectively (Day 7 blastocysts: total N = 14, Day 8 blastocysts: total N = 14, Day 9 blastocysts: total N = 10). The fluorescent intensities of each image were digitized using Image J software (National Institutes of Health, Bethesda, MD, USA), and the ratio of red to green fluorescence was observed.

### Quantitative reverse transcription polymerase chain reaction to study bovine blastocysts’ gene expression

Total RNA was extracted from a single snap-frozen blastocyst using an RNeasy Micro Kit (Qiagen GmbH, Hilden, Germany) following the manufacturer’s instructions (total N in each group = 14). The yield of total mRNA was not quantifiable, as the amount was too low. First-strand cDNA was synthesized from all the mRNA eluted from the RNA extraction step using HiSenScript RH(−) RT PreMix Kit (iNtRON Biotechnology, Inc., Sungnam, Korea). Gene expression of blastocysts was explored by quantitative reverse transcription polymerase chain reaction (qRT-PCR) using a Rotor-Gene Q qRT-PCR instrument with a QuantiNova SYBR Green PCR kit (both Qiagen GmbH), including 0.7 μM forward and reward primers under the amplifying conditions of pre-denaturation at 95°C for 2 minutes, and 40 PCR cycles at 95°C and 60°C for 5 and 10 seconds, respectively. The PCR for each primer was executed once only because of the limited quantity of cDNA. Rotor-Gene Q Series Software (Qiagen GmbH) was employed to obtain the cycle threshold values, followed by normalization of the target genes for early embryonic development (*Oct4*, *Sox2*, and *Cdx2*), apoptosis (*Bax* and *Bcl2*), and mitochondrial activity (*Cox1* and *Tfam*) against a reference gene (*Gapdh*). The primers’ information is shown in Table 1.

### Statistical analysis

The values between the three groups (Day 7, 8, and 9 blastocysts) were assessed for normal distribution with equal variance and statistically analyzed using one-way analysis of variance (ANOVA) with Tukey’s *post hoc* test using SPSS software v.12.0 (IBM Statistical Software; IBM Corp., Armonk, NY, USA). In addition, Pearson’s correlation analysis was conducted between several factors (the ICM and apoptosis ratio) and the pregnancy rate. Differences were considered statistically significant at p<0.05. The graphs are presented as the mean±standard error of the mean.

## RESULTS

### Early embryo development yields and morphological observations

**Figure 1:**
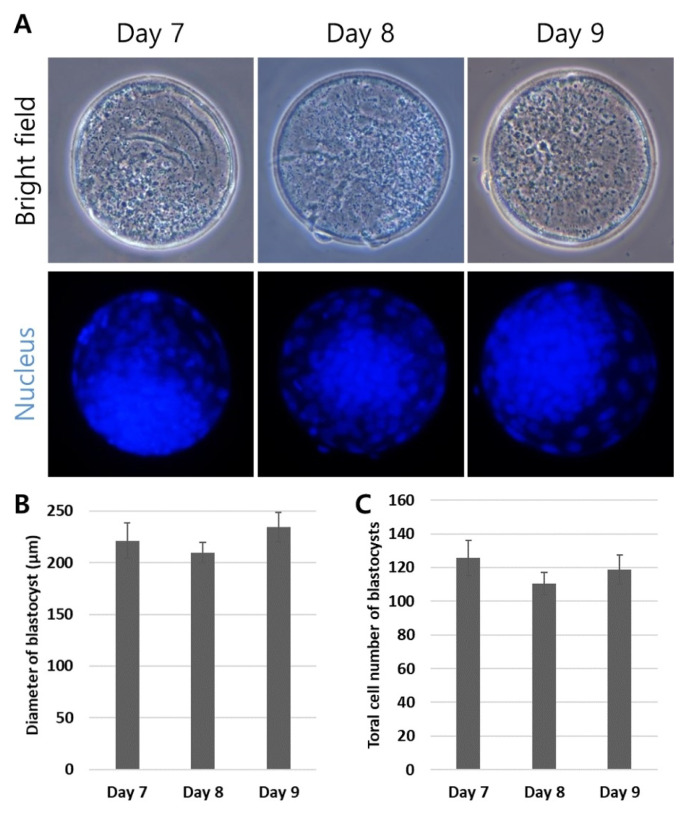
Morphological observation of the *in vitro* fertilized embryos. (A) Representative images of the Day 7, 8, and 9 blastocysts. (B) The mean diameters did not differ between the Day 7, 8, and 9 blastocysts. (C) The difference in total cell numbers was determined by counting the 4′,6-diamidino-2-phenylindole (DAPI)-positive cells and was not significantly different between the Day 7, 8, and 9 blastocysts. Graphs are presented as the mean±standard error of the mean. Magnification: ×400.

Table 2 shows that the overall Day 3 cleavage and total blastocyst formation rates were 81.0±3.8% and 31.7±2.3%, respectively. The Day 9 blastocyst formation rate (7.4±0.8%) was significantly lower (p<0.05) compared with the Day 7 and 8 blastocysts’ formation rates (13.0±0.9% and 11.5±0.7%, respectively). There were no unusual or notable differences in the morphological observations between the Day 7, 8, and 9 blastocysts (Figure 1A). In addition, the blastocysts’ total cell numbers and the diameter of each group’s cells were not significantly different (Figure 1B). This demonstrated that the morphological features were similar between the Day 7, 8, and 9 blastocysts.

### Pregnancy post-embryo transfer of blastocysts with different developmental speed

**Figure 2:**
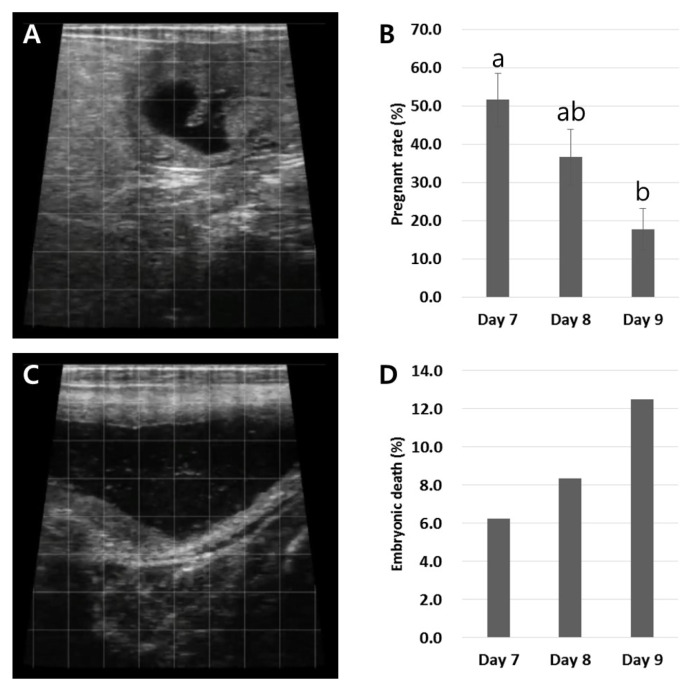
Pregnancy post-embryo transfer (ET) of blastocysts with different developmental speed. (A) A pregnancy diagnosis was conducted at 30 days post-ET by checking for the presence of the gestational vesicle. (B) The pregnancy rate significantly reduced (p<0.05) in the Day 9 blastocysts compared with the Day 7 blastocysts. (C) Early embryonic death was determined by transrectal ultrasonography on day 60 post-ET. (D) The Day 9 blastocyst recipients exhibited greater pregnancy loss. Graph B or D is presented as the mean±standard error of the mean or the mean, respectively. Significant differences are indicated by different letters on the top of the bars (p<0.05).

A total of 166 blastocysts from 14 batches were transferred into the recipients and the results of the pregnancy diagnosis at 30 and 60 days post-ET are shown in Figure 2. Pregnancy diagnosis after 30 days of ET showed the rate of pregnancy in the Day 7 blastocyst group was more than 50% (51.7±7.0%; total N of pregnant heifers/total recipients = 48/90). There was a decrease in the pregnancy rate in the Day 8 blastocyst group (36.7±7.3%; total N of pregnant heifers/total recipients = 14/40) compared with the Day 7 blastocysts; however, the difference was not significant. A significant decrease (p<0.05) in pregnancy was observed in the Day 9 blastocyst group (17.8±5.5%; total N of pregnant heifers/total recipients = 8/36), compared with the Day 7 blastocyst group (Figure 2B). At 60 days post-ET, when all experimental groups were combined, the overall pregnancy loss rate was 7.1% (total N of pregnancy loss/pregnancy = 5/70) and higher prevalence was presented in the Day 9 blastocysts (12.5%; N of pregnancy loss/pregnancy = 1/8); however, this finding may be unreliable because of its small frequency (Figure 2D).

### Early embryonic development assessment in blastocysts with different developmental speed

**Figure 3:**
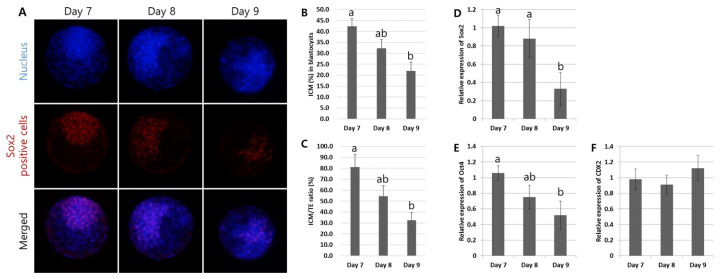
Assessment of early embryonic development in blastocysts with different developmental speed. (A) Differential immunofluorescent staining to identify the inner cell mass (ICM) was conducted on the Day 7, 8, and 9 blastocysts. (B) Based on the number of total cells (4′,6-diamidino-2-phenylindole [DAPI]-positive, blue) and ICM cells (*Sox2*-positive, red), the ratio of the ICM in the blastocysts was calculated. The Day 9 blastocyst recipient group presented a significant decrease (p<0.05) in the ICM (%) compared with that of the Day 7 blastocyst group. (C) The ICM/TE (%) (number of ICM cells versus TE [*Sox2*-negative] cells) showed a significantly lower ratio (p<0.05) in the Day 9 blastocyst group compared with that of the Day 7 group. (D–F) The gene expression study for early embryo development demonstrated that the pluripotent transcriptional factors *Sox2* and *Oct4* were significantly decreased (p<0.05) in the Day 9 blastocyst group compared with those of the Day 7 group; however, the TE marker (*Cdx2*) was not significantly different between the groups. TE, trophectoderm. Magnification: ×400. Graphs are presented as the mean±standard error of the mean. Significant differences are indicated by different letters on the top of bars (p<0.05).

To assess differences in the early embryonic development of the Day 7, 8, and 9 blastocysts, differential staining to assess the ICM and early embryo development-related gene expression studies were performed (Figure 3). Immunofluorescent staining for *Sox2*, a specific marker of ICM in cows, showed its expression in all the blastocysts (Figure 3A). However, the cell number of the ICM (%) that was a *Sox2*-positive population gradually and significantly decreased from the Day 7 (42.4±3.4%) to the Day 9 blastocysts (22.0±3.9%) (p<0.05) (Figure 3B). In addition, the ratio of ICM/TE (*Sox2*-negative cells) showed a similar significance pattern (p<0.05) (Figure 3C). In the gene expression study, the blastocysts’ early embryo developmental gene expression (*Sox2* and *Oct4*) was significantly higher (p<0.05) in the Day 7 blastocysts compared with the Day 9 blastocysts (Figures 3D, 3E). In contrast, the expression of the TE-specific gene (*Cdx2*) expression did not differ between the groups. These findings indicated that late-developing blastocysts (Day 9 blastocysts) had attenuated ICM formation ability, which was more important in the fetus’ definitive structures compared with the early-developing embryos.

### Measurement of apoptosis in the blastocysts with different developmental speed

**Figure 4:**
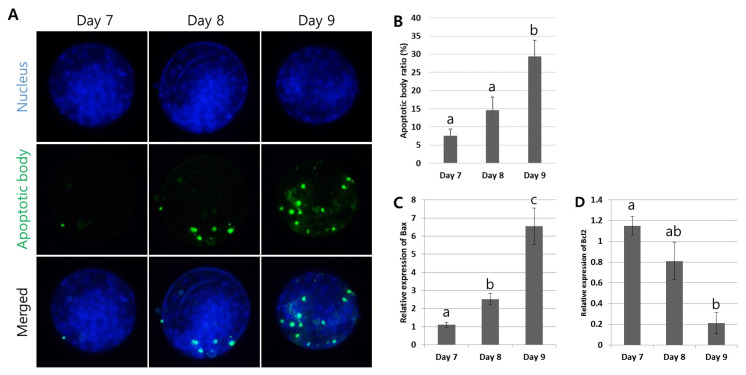
Measurement of apoptosis in blastocysts with different developmental speed. (A) The terminal deoxynucleotidyl transferase dUTP nick end labeling (TUNEL) assay was used to identify apoptotic bodies in the Day 7, 8, and 9 blastocysts. (B) Based on the number of total cells (4′,6-diamidino-2-phenylindole [DAPI]-positive, blue) and apoptotic bodies (TUNEL-positive, green), the ratio of apoptotic cells (%) in the blastocysts was assessed. A significant elevation (p<0.05) in apoptotic cells (%) was found in the Day 9 blastocyst group, compared with the Day 7 and 8 blastocyst groups. (C and D) The gene expression study presented a significant increase (p<0.05) in pro-apoptosis (*Bax*) but a decrease in anti-apoptosis (*Bcl2*) in the Day 9 compared with the Day 7 and 8 blastocyst groups. Magnification: ×400. Graphs are presented as the mean±standard error of the mean. Significant differences are indicated by different letters on the top of bars (p<0.05).

Apoptosis is an important indicative tool for determining embryo competence. For the comparative analysis of apoptosis in the Day 7, 8, and 9 blastocysts, the TUNEL assay was used to visualize DNA fragmentation and gene expression for pro- or anti-apoptosis was studied (Figure 4). The TUNEL assay revealed that most of the blastocysts possessed apoptotic bodies (Figure 4A). However, the number of apoptotic bodies in the total cell (%) was significantly higher (p<0.05) in the Day 9 blastocysts (29.4±4.4%), compared with the Day 7 (7.6±1.9%) and Day 8 (14.6±3.6%) blastocysts (Figure 4B). In the gene expression study, the different developmental speed of the blastocysts showed that the mRNA for pro-apoptosis (*Bax*) significantly increased (p<0.05) from the Day 7 to Day 9 blastocysts; in contrast, the anti-apoptosis-related gene (*Bcl2*) expression was inversely proportional (Figures 4C, 4D). These observations implied that cells in the late-developing embryos were more prone to programmed cell death.

### Mitochondrial membrane potential in blastocysts with different developmental speed

**Figure 5:**
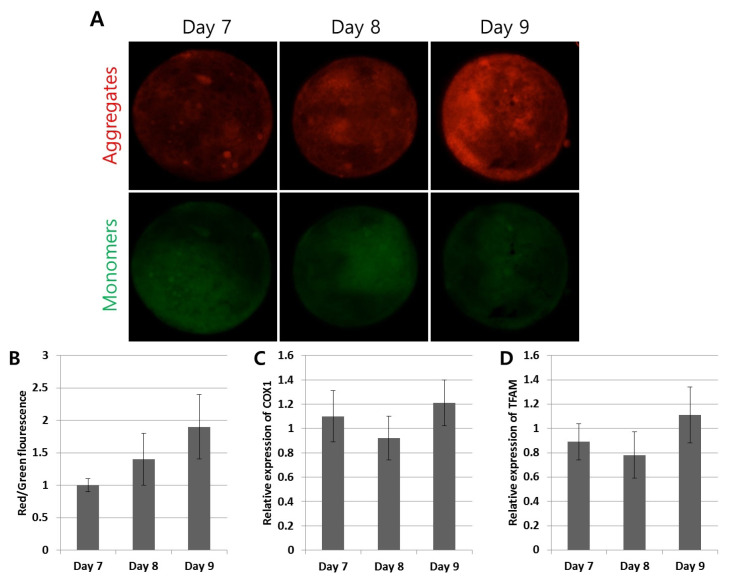
Assessment of mitochondrial membrane potential in blastocysts with different developmental speed. (A) The JC1 staining to investigate the changes in the mitochondrial membrane potential in Day 7, 8, and 9 blastocysts. The monomers (low mitochondrial membrane potential) and aggregates (high mitochondrial membrane potential) were heterogeneously distributed over the blastocysts. (B) There were no significant changes in the ratio of red to green signal intensity between the three blastocyst groups. (C and D) The gene expression study did not identify considerable changes in the mitochondrial activity-related genes (*Cox1* and *Tfam*). Magnification: ×400. Graphs are presented as the mean±standard error of the mean.

To investigate the changes in Day 7, 8, and 9 blastocysts’ mitochondrial membrane potential to generate ATP, JC1 staining was used (Figure 5). The green (monomers as low mitochondrial membrane potential) and red (aggregates as high mitochondrial membrane potential) signal areas were heterogeneously distributed over the blastocysts (Figure 5A). When the red signal (aggregates) was compared relative to the green signal (monomers), there was an increased tendency from the Day 7 to Day 9 blastocysts but this was not significant (Figure 5B). In addition, the mitochondrial activity-related genes (*Cox1* and *Tfam*) did not present any changes across the blastocysts with different developmental speed. These findings indicated that the mitochondrial membrane potential and activity were maintained, even in late-developing blastocysts.

### Correlation analysis between results derived from assays and pregnancy rate

**Figure 6:**
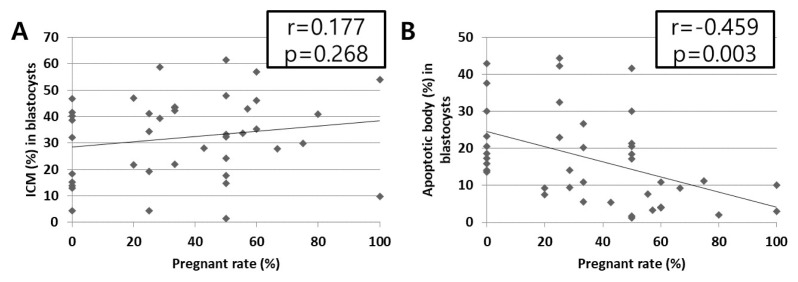
Correlation analysis between the blastocysts’ inner cell mass (ICM) and apoptosis and the pregnancy rate. (A) The correlation between the ICM (%) and pregnancy rate (%) was not significant. (B) A high and significant negative correlation (p<0.05) was seen between the population of apoptotic cells (%) and the pregnancy rate (%).

The results of this study indicated that late-developing (Day 9) blastocysts presented a significant decrease (p<0.05) in the pregnancy rate (Figure 2), a reduction in the ICM (%) (Figure 3), and an elevation of apoptotic cells (%) (Figure 4), relative to those of the Day 7 and 8 blastocysts. Pearson’s correlation assay was performed (Figure 6) to identify possible reasons for decreased pregnancy rates in the late-developing blastocysts. The correlation between the ICM (%) and the pregnancy rate was not significant (p = 0.268; r = 0.177) (Figure 6A). In contrast, the pregnancy rate presented a highly and significantly negative correlation with the population of apoptotic cells (p = 0.003; r = −0.459), indicating that the embryo competence of Day 9 blastocysts for successful conception was directly related to their apoptosis level (Figure 6B).

## DISCUSSION

ET in cattle is becoming increasingly important globally, especially to produce genetically superior offspring and maximize economic benefits [19]. Numerous studies have focused on increasing ET efficiency, as regards the quality of embryos, improving pregnancy rates, and a control strategy for breeding management. Among these factors, producing high-quality embryos is the most fundamental and crucial factor to guarantee successful ET. The mortality of fertilized embryos is sustained within the first 2 to 3 weeks; consequently, high-quality embryos may increase successful implantation and pregnancy post-ET, reducing the rate of developmental failure [11]. Fertilized oocytes typically develop toward blastocysts between days 6.5 to 10; therefore, a comparative assessment of blastocysts at different developmental speed, that is, early to late-developing blastocysts, is necessary to establish a clear strategy for achieving high-quality embryos capable of higher implantation and pregnancy rates in ET recipients. Therefore, this study compared IVF blastocysts with different developmental speed that first appeared on Day 7 (early-developing blastocysts), and Day 8 and 9 (late-developing blastocysts) post-IVF. While there was no outstanding difference in the blastocysts’ morphological characteristics, immunoassays with *Sox2* and TUNEL demonstrated that the late-developing blastocysts presented lower ICM formation and increased apoptotic cells, compared with the early-developing blastocysts.

Previously published literature about bovine IVF embryos indicates that approximately 80% of fertilized oocytes are cleaved post-IVF, about 30% of fertilized oocytes reach the blastocyst stage, and the pregnancy rate after ET is between 30% and 65% [3,6–8,19,20]. This study’s results were also similar to the reported embryo developing potential, including 81.0% cleaved rates, 31.7% total blastocyst formation, and 51.7% to 36.7% pregnancy rates for Day 7 to 8 blastocysts and 17.8% pregnancy rates for Day 9 blastocysts (Table 2; Figure 2). Similar studies regarding blastocyst developmental speed-dependent pregnancy rates have demonstrated that Day 7 blastocysts exhibit a higher pregnancy rate (49.0%) compared with late-developing blastocysts at Day 8 (36.4%) and Day 9 (15.4%) in Hanwoo cattle [6]. Demetrio et al [8] found that Day 6 early-developing embryos achieved a higher pregnancy rate compared with Day 7 to 8 embryos. Furthermore, when Day 7 blastocysts were transferred, the pregnancy rate was even lower if they had not reached the blastocyst stage at Day 6 or the expanded blastocyst stage at Day 7. And embryos with any type of developing stage (morula and early to hatched blastocysts) at Day 7 had more successful pregnancy rates post-ET compared with the corresponding stages of Day 8 blastocysts [10]. However, the mechanisms and molecular features explaining why late-developing blastocysts presented lower pregnancy rates are still unclear. Therefore, this study aimed to identify the molecular features that influence the pregnancy ability of blastocysts with different developmental speed. The results demonstrated that a gradual increase of apoptosis from early to late-developing blastocysts (Figure 4) was negatively correlated with a lower pregnancy rate (Figure 6).

Fertilized oocytes require highly complex and orchestrated gene expression for normal development toward blastocysts. The maternal transcripts support early zygote development; then, the activated embryonic genome initiates protein synthesis for embryo development (8 to 16 cell stage in bovine). Therefore, the successful control of temporal and spatial gene expression is vital to early embryo development. During early embryo development, the outer cells of the embryo are differentiated into TE cells by regulating *Cdx2* expression; in contrast, the inner cells preserve pluripotent capacity, self-renewal, and cell-to-cell communication through gap junctions by pluripotent gene expression [21]. Therefore, pluripotent genes, including *Oct4*, *Sox2*, and *Nanog*, play a vital role in normal preimplantation embryo development and their down-regulation may decrease embryo developmental competence. The expression of *Oct4* in bovine blastocysts has been related to better morphology, increased total cell numbers, and the regulation of *Cdx2* gene expression [22]. In addition, *Oct4* is detected from the oocyte stage but decreases post-fertilization as the maternal transcripts is depleted; this is followed by an increase from the morula stage after embryonic genome activation [23]. However, it has been suggested that *Oct4* is not an ICM-specific marker in cattle; this is because the cell number in parthenogenetic embryos does not reduce with decreased *Oct4* expression [24], and *Oct4* has been found in both ICM and TE cells [13]. Consistent with *Oct4*, *Sox2* is an essential gene to preserve the self-renewal ability and undifferentiated state of bovine ICM; decreased expression induces poor quality blastocysts with less total cell numbers and reduced Nanog expression [13,14]. Moreover, *Sox2* expression is initiated from the metaphase II oocyte stage and continues until the cleaved embryo stage. However, in contrast to *Oct4* expression in blastocysts, *Sox2* expression is restricted to the ICM and it can, therefore, be a specific maker for bovine blastocysts’ ICM [13,14]. Another key gene during early embryo development is *Cdx2*, which plays a role in regulating the formation and functioning of the TE. The transcription factor *Cdx2* is primarily distributed in the TE and weakly in the ICM of the bovine blastocysts [23]. In addition, *Cdx2* is highly related to the success of ET; a fertile blastocyst to calf delivery exhibited enriched *Cdx2* expression [11]. Similar to *Oct4* expression, the maternal *Cdx2* transcript has been reported to decrease after fertilization, followed by an increase from the morula stage and throughout the entire period of blastocyst development [13,23]. However, *Cdx2* expression is inversely proportional to *Oct4* transcription at the time of TE formation during the blastocyst’s differentiation [25]. This study’s results indicated that *Oct4* and *Sox2* expression was lower in the Day 9 blastocysts compared with the Day 7 blastocysts (Figure 3), which implied that the self-renewal ability and undifferentiated state of the bovine ICM were slowly weakened in the late-developing blastocysts. Therefore, this study checked the *Sox2*-positive cell population as the number of the ICM cells to investigate if the quality changes depended on the developmental speed toward blastocysts; this showed that smaller ICM cell numbers resulted in late-developing embryos. Because early- and late-developing blastocysts were not morphologically different in size and total cell number (Figure 1), the smaller ICM/TE ratio (Figure 3) implied that complex and orchestrated gene expression for normal embryo development was not well progressed in late-developing blastocysts.

All cells undergo apoptotic cell death to balance between cell survival, proliferation, and death. In embryos, apoptosis in the ICM is necessary to maintain their cellular quality by removing damaged, excessive, non-required, and developmentally incompetent cells, causing the ICM cell population to reach a plateau [26]. Apoptosis is mainly regulated by *Bcl2* gene family members, comprising anti-apoptotic genes (e.g., *Bcl2*) to prevent apoptosis and pro-apoptotic genes (e.g., *Bax* and *Bak*) to trigger apoptosis [14]. In particular, the ratio of *Bcl2* to *Bax* is a critical determinant of either cell survival or death and unbalanced expression can induce cell death and apoptosis [14,27]. Pro- and anti-apoptotic genes are also key regulators in embryos and are consistently observed during all the early embryo development stages [28]. The literature reports that gene expression patterns for increased pro-apoptosis with decreased anti-apoptosis are directly associated with poor-quality embryos [14,20]. However, the up- and down-regulation of these genes are embryo development stage-dependent; expression of the anti-apoptotic genes is dominant from the oocyte to the 16-cell stage during early embryo development and apoptosis-related gene expression is higher after the 16-cell stage [29]. In the case of the relationship between embryo quality and apoptosis, a highly negative correlation has been reported between the blastocyst formation rate and apoptosis, and a positive correlation between the pregnancy rate and *Bcl2* mRNA transcripts has been identified [27]. In terms of embryo grade and apoptosis, grade 4 oocytes exhibit more typical features of apoptosis post-IVF compared with grade 1 oocytes [14]. Regarding the relationship between apoptosis and the different developmental speed of blastocysts, the population of apoptotic cells in blastocysts, determined by the TUNEL assay, and the expression of the pro-apoptosis gene *Bax* were higher in Day 8 compared with Day 7 blastocysts [15,30]. Likewise, this study aimed to expand on previously reported results by comparing the apoptosis levels of early to late-developing embryos, that is, Day 7, 8, and 9 blastocysts; the results showed an increase in the number of apoptotic bodies in the total cells of late-developing blastocysts. In addition, the ratio of pro-apoptosis (*Bax*) to anti-apoptosis (*Bcl2*) gene expression was more evident in late-developing compared with early-developing blastocysts (Figure 4). The accumulated results from this and previous studies support that late-developing blastocysts have lower fertility to pregnancy because of low-quality embryos that exhibit high apoptosis despite appearing morphologically normal (Figure 6).

Mitochondria in the cytoplasm of oocytes are maternally inherited and play a pivotal role in several cellular functions, including ATP production to provide energy for fertilization and preimplantation development, reactive oxygen species generation, apoptosis control, and calcium and iron homeostasis [17]. Therefore, the mitochondria’s function and activity are thought to be the essential determinants of alive cells, including early embryos, which need cellular spindle movement, chromosome duplication and segregation, regulation of the cellular cycle, and developmental morphological changes in the blastocysts, such as compaction, cavitation, and hatching [17]. In addition, during normal maturation of oocytes (meiosis), the mitochondria are redistributed from being homogenous to heterogeneous in the nucleus-enriched area via the cytoskeletal network. In addition, healthy and normally developing embryos present a higher ATP content with mitochondrial activity than abnormal and arrested embryos [4]. When bovine IVF embryos were comparatively investigated during various developing stages from zygotes to blastocysts for mtDNA-related gene quantification, the gene expression patterns were variable. Expression of *Cox1* and *Tfam* was low or undetectable at the zygote stage but increased sharply at the morula stage and throughout the entire period of blastocyst development, suggesting *de novo* synthesis [17]. In a study of mtDNA and mitochondrial function on bovine embryo competence, high-quality embryos demonstrated a tendency for more mtDNA molecules in oocytes compared with low-quality embryos [16]. To determine the relationship between mitochondrial membrane potential and activity on bovine blastocysts with different developmental speed, this study conducted JC1 assays and relevant gene expression studies (Figure 5). All Day 7, 8, and 9 blastocysts exhibited heterogeneously distributed monomers (low mitochondrial membrane potential) and aggregates (high mitochondrial membrane potential) but the ratio of monomers to aggregates was not significantly different between the groups. In addition, mtDNA-related genes (*Cox1* and *Tfam*) were not differently expressed in the blastocysts with different developmental speed. These findings suggest that blastocyst-formable embryos, regardless of their different developmental speed, have normal mitochondria function, even in late-developing blastocysts.

## CONCLUSION

Diverse internal and external factors have complex effects on embryo quality during *in vitro* production and their fertility to pregnancy. Consequently, it is difficult to identify a single cause or factor that determines an embryo’s success or failure of pregnancy post-ET. Therefore, this study focused on determining how the differences between blastocysts with different developmental speed at the molecular level (i.e., morphology, early embryo development, apoptosis, and mitochondrial activity) influenced their *in vivo* application (i.e., ET and pregnancy rate). The results indicated that late-developing embryos, despite their normal morphology, had impaired fertility to pregnancy rates compared with early-developing blastocysts; this can be attributed to lower embryo quality primarily from increased apoptosis. This study’s results may contribute to establishing an improved strategy for bovine ET in the field of animal bioscience and biotechnology.

## Figures and Tables

**Table 1:** Primer information for gene expression study in bovine blastocysts

Gene	Forward	Reward	BP
*Oct4*	CAAATTAGCCACATCGCC	AGCCTCAAAATCCTCACG	126
*Sox2*	TCAGATGCAGCCCATGCAC	GGTGCCCTGCTGAGAATAGGAC	121
*Cdx2*	AGTGAAAACCAGGACGAAAGA	CTCTGAGAGCCCCAGCGT	142
*Bax*	CTCCCCGAGAGGTCTTTTTC	TCGAAGGAAGTCCAATGTCC	176
*Bcl2*	TCGTGGCCTTCTTTGAGTTC	CGGTTCAGGTACTCGGTCAT	109
*Tfam*	CAAATGATGGAAGTTGGACG	AGCTTCCGGTATTGAGACC	148
*Cox1*	CCCAAACTGAGCACATGGC	GTTAAGTATGTCTGAATCGTC	162
*Gapdh*	TTCAACGGCACAGTCAAGG	ACATACTCAGCACCAGCATCAC	119

BP, base pair.

**Table 2:** Developmental efficiency of bovine IVF embryos

COCs	Cleaved zygotes (%)	Total blastocyst formation (%)	Day 7 blastocyst formation (%)	Day 8 blastocyst formation (%)	Day 9 blastocyst formation (%)
1,367	1,105 (81.0± 3.8)	434 (31.7±2.3)	177 (13.0±0.9)^a^	156 (11.5±0.7)^a^	101 (7.4±0.8)^b^

Values (%) are presented as the mean±standard error of the mean.

IVF, *in vitro* fertilization; COCs, cumulus-oocyte complexes.

a,b Blastocyst formation rates between the Day 7, 8, and 9 blastocysts with different superscripts are significantly different (p<0.05).
